# The neural mechanisms underlying the processing of consonant, vowel and tone during Chinese typing: an fNIRS study

**DOI:** 10.3389/fnins.2023.1258480

**Published:** 2023-12-19

**Authors:** Jianan Yu, Yun Zou, Yan Wu

**Affiliations:** ^1^School of Psychology, Northeast Normal University, Changchun, Jilin, China; ^2^Department of Psychological and Brain Sciences, University of Massachusetts Amherst, Amherst, MA, United States

**Keywords:** Chinese character production, fNIRS, consonant, vowel, tone

## Abstract

Many studies have explored the role of consonant, vowel, and tone in Chinese word identification or sentence comprehension. However, few studies have explored their roles and neural basis during Chinese word production, especially when involving neural basis. The present fNIRS study investigated the neural mechanisms of consonant, vowel, and tone processing during Chinese typing. Participants were asked to name the Chinese characters displayed on a computer screen by typing on a keyboard while hearing a simultaneously presented auditory stimulus. The auditory stimulus was either consistent with the characters’ pronunciation (consistent condition) or mismatched in the consonant, vowel, or tone of the character pronunciation. The fNIRS results showed that compared with the consistent condition (as baseline), the consonant mismatch condition evoked lower levels of oxygenated hemoglobin (HbO) activation in the left inferior frontal gyrus Broca’s triangle and left superior temporal gyrus. Vowel mismatch condition evoked a higher level of HbO activation in the top of the left inferior frontal gyrus and left middle frontal gyrus. The regions and patterns of brain activation evoked by tone mismatch were the same as those of vowel mismatch. The study indicated that consonant, vowel and tone all play a role in Chinese character production. The sensitive brain areas were all in the left hemisphere. However, the neural mechanism of consonant processing differed from vowel processing in both brain regions and patterns, while tone and vowel processing shared the same regions.

## 1 Introduction

Spoken language consists of both segmental and super-segmental information. Segmental information includes vowels and consonants, while super-segmental information includes stress, prosody, and tonal information. If tonal information is used only to express the emotional feelings of the speaker, then the language is considered non-tonal language, such as English, German, and French. However, in more than 40% of languages, word meanings were constrained by tonal information (Jia et al., 2015). These languages are called tonal languages (Pike, 1948). For example, Mandarin Chinese is a tonal language with four tones. The words “戏剧” (drama) and “喜剧” (comedy) have the same segmental information in Chinese, namely “xi-ju,” but their tonal information is different (戏剧: xi4-ju4; 喜剧: xi3-ju4), resulting in different pronunciations and meanings. Given the importance of tonal information in Chinese, the relative role of tonal and segmental information in tonal language processing has attracted considerable attention from researchers (Ye and Connine, 1999; Gandour et al., 2003; Tong et al., 2008; Malins and Joanisse, 2010; Hu et al., 2012; Li et al., 2013, 2014; Zhao et al., 2016; Choi et al., 2017; Zou et al., 2020; Zhang et al., 2021).

Language processing includes both comprehension and production. The respective roles of tone and segment have been a focus in speech comprehension studies in tonal languages. It is generally believed that segmental information, particularly vowel, is more crucial than tonal information in constraining word identity (Tong et al., 2008; Li et al., 2013; Zhao et al., 2016). However, little is known about the role of segmental and tonal information in language production. Most existing studies manipulated either segmental information (Wong and Chen, 2009; Zhang et al., 2011; Wong et al., 2012, 2018; Wang et al., 2018) or tonal information (Zhang et al., 2007; Chang et al., 2014; Chang and Kuo, 2016) to investigate how each of them solely contributes to speech production. Few studies manipulated both and compared their relative roles in language production (Liu et al., 2006; Wong and Chen, 2008, 2015; Zhang and Damian, 2009; Wang et al., 2015; Wiener and Turnbull, 2016). To fill this gap, this study using fNIRS technology will manipulate both segmental (consonant and vowel) and tonal information to explore their relative roles and the neural mechanisms of tone and segment processing during language production.

A commonly used paradigm to examine the role of tone and segment in speech processing is the picture-word-interference (PWI) paradigm (Wong and Chen, 2008, 2009, 2015; Wang et al., 2018; Wong et al., 2018; Hitomi et al., 2021; Zhang et al., 2022). In this paradigm, participants need to name certain pictures presented simultaneously with distractors. The effects of the distractors on picture naming depend on the relationships between the distractors and the target words. It was found that the distractors (e.g., dog) can inhibit the target word naming (e.g., cat) if they belong to the same semantic category. This effect was known as the semantic interference effect, which might result from the competition between targets and distractors in lexical selection. Conversely, the distractors having phonological overlap with the targets (e.g., cap) can facilitate target naming (e.g., cat). This effect was known as the phonological facilitation effect, which might be due to the cross-modal priming effects of the distractors on phonological encoding during target naming. More precisely, hearing the shared phonological units in the distractors might enhance the activation of those sub-syllabic units during target word naming, which further facilitates the phonological encoding of the targets (Bi et al., 2009). By comparing the phonological facilitatory effects induced by the distractors that shared the tonal or segmental information with the targets, researchers could infer the relative roles of segments and tones during Chinese spoken word production.

Using PWI paradigm, Wong et al. (2018) investigated how tonal and segmental information contribute to Cantonese spoken word production. Participants were asked to name pictures meanwhile being presented with visual or spoken monosyllabic words as distractors. The distractors were either completely irrelevant to the pictures’ intended names (as baseline) or shared the consonant, vowel, tone, consonant-plus-vowel, consonant-plus-tone, and vowel-plus-tone as the pictures’ intended names. In the case of visual distractors, the authors found that the words sharing only the consonant, vowel, or tone could not facilitate picture naming. However, the words sharing consonant-plus-vowel or vowel-plus-tone produced significant faciliatory effects. It was speculated that individual segment or tone could not produce robust effects in Cantonese spoken word production, particularly when the distractors were the visual monosyllabic words (i.e., characters) that have only weaker connections between word forms and pronunciations (Chen et al., 2009). Nevertheless, when the auditory distractors were used, the spoken words sharing only the vowel with the pictures’ intended names produced significant facilitatory effects, while the spoken words sharing only the tone or consonant produced null effects, indicating a more crucial role of vowel while weaker effects of consonant and tone during Cantonese spoken word production. The more important role of vowel and weaker role of tone or consonant in Chinese spoken word processing were also found in Wiener and Turnbull (2016) and Wong and Chen (2015).

In addition to using behavioral methods, some researchers have explored the processing of segments and tones in language production from a cognitive neuroscience perspective (Liu et al., 2006; Zhang and Damian, 2009). For example, using the fMRI technique with an adaptation paradigm, Liu et al. (2006) investigated the neural correlates of vowels and tones processing in speech production. The experiment had two conditions: constant vowel with changing tone and constant tone with changing vowel. The consonant in both conditions was “sh.” Participants were asked to name the characters or pinyin presented on a screen. The results showed that both tone and vowel changes activated the bilateral inferior frontal gyrus, insula, and anterior central gyrus, with greater activation in the left brain than the right. Additionally, the study found that compared with vowel change, tone change resulted in greater activation in the right inferior frontal gyrus. This study suggested that both vowels and tones play a role in Chinese production and involve similar neural bases. However, tone processing is stronger than vowel processing in the right brain. Therefore, there is a discrepancy between the results from neurological and behavioral studies regarding the role of tonal information in speech production. By comparing a tone judgment task (the participants judged whether each pair of two Chinese characters carried the same lexical tone) with a baseline condition (the participants just relaxed with no overt response required), Kwok et al. (2016) found that the tone judgment task evoked greater activation in the bilateral frontal lobe and left parietal lobe, which again reflect the involvement of tone in Chinese word processing.

It is worth noting that the above findings mainly focused on speech production. However, language output includes not only speech production but also handwriting, body language, and typing, etc. Compared to body language and typing, handwriting has been studied more extensively (Longcamp et al., 2003; Cohen et al., 2004; James and Gauthier, 2006; Rapp and Dufor, 2011; Dufor and Rapp, 2013; Purcell and Rapp, 2013; He and Zhang, 2017; Wang and Zhang, 2021). However, few researchers have studied the role of consonants, vowels, and tones in other language output modes besides speech production. Speech is still the primary mode of language output in daily life, but with the development of society, people’s communication increasingly relies on the Internet. As a result, typing has become a mainstream mode of communication (Zhu et al., 2009). In daily life, traditional pen-and-paper writing has gradually been replaced by typing, even in primary education (Longcamp et al., 2005). Although some studies have found that excessive use of typing may lead to a decline in handwriting speed and fluency and deterioration of handwriting motor skills (Sülzenbrück et al., 2011), typing does have tremendous advantages in other aspects. For example, typing is much faster than writing, and the content of typing is more recognizable (Chen et al., 2016a,b).

Therefore, typing has received increasing attention in recent studies. Some studies have found that the cognitive processes underlying typing in English is similar to the handwriting system but different from the speech system. This was attributed to the consistency of the English typing and handwriting processes since they both require spelling out correct words directly through letter selection and arrangement, involving phonetic and orthographic processing of words (Ouellette and Tims, 2014; Chen et al., 2016a,b). According to this logic, the cognitive processes involved in typing should be more similar to speech production than handwriting in Chinese because the pinyin input method used for typing relies more on phonetic spelling ability than handwriting due to the low correspondence between orthographic and pronunciation (Chen et al., 2009). Evidence for this speculation was found in a Chinese study by Zhu et al. (2009), which showed that proficient typing participants had higher pronunciation consistency judgment ability than poor typing participants. However, the two groups did not differ in orthographic consistency judgment ability. Based on the results, the authors speculated that pinyin typing might strengthen the connection between phonetics and semantics and improve the sensitivity to speech. A meta-analysis (Lyu et al., 2021) also showed that typing had a greater impact on phonological recognition in Chinese learners than handwriting. This effect is more prominent in Chinese than in English. Therefore, with the development of typing as a mainstream mode of communication, typing has played an increasingly important role in the development of individual’s language ability. As mentioned above, typing can enhance the phonological-semantic connection. However, the role of consonant, vowel and tone in typing and the neural basis of their effects remain to be explored.

To fill this gap, the present study aims to take typing as the research object to explore the role and neural basis of consonant, vowel, and tone in language production using fNIRS technique. Compared with other neurological research methods such as EEG and MEG, the fNIRS technique is more suitable for exploring the process of language production because its data acquisition process is more resistant to interference from the muscle moves during language generation processes (Quaresima et al., 2012). In addition, fNIRS equipment produces very little noise during operation, ensuring that participants can complete the experiment in a relatively natural environment with high ecological validity (Ferrari and Quaresima, 2012).

This study draws on the PWI paradigm and adopts the word-word interference (WWI) paradigm. Participants were asked to perform a naming task on visually presented Chinese characters under the influences of auditory distractors. By manipulating the auditory interference, we set up four conditions: consistent, consonant mismatch, vowel mismatch, and tone mismatch. In this way, we examined the neural responses underlying the processing of consonant, vowel, and tone during typing by comparing each mismatch condition with the consistent condition. Based on the previous studies (Liu et al., 2006; Hu and Xu, 2011; Chang et al., 2014; Chang and Kuo, 2016), we expect that consonants, vowels, and tones will all play a role in language production. However, their functions and neural associations will be different, among which the tone may be lateralized in the right hemisphere (Liu et al., 2006).

In this study, we also measured participants’ phonological awareness through a phonemic deletion task to explore whether individual differences in phonological awareness would affect the processing of consonant, vowel, and tone. The issue of individual differences has become increasingly important in the field of language processing (Kidd et al., 2018). The development of language processing theory cannot be separated from the exploration of individual differences. Phonological awareness refers to an individual’s ability to pronounce Chinese characters or use phonemes (Wu et al., 2020). Previous studies have found that phonological awareness is closely related to human language comprehension ability (Li et al., 2011). Stronger phonological awareness is associated with better development of individual reading comprehension ability. However, whether phonological awareness also correlates with language production remained unclear. We hypothesized that phonological awareness should be related to the processing of consonant, vowel, and tone during language production. However, the exact relation between phonological awareness and consonant, vowel and tone processing remain to be further explored.

## 2 Method

### 2.1 Participants

In this experiment, 28 participants were recruited. All participants were right-handed, with normal or corrected-to-normal vision and hearing, and no history of psychiatric disorders or participation in similar experiments. Written informed consent was obtained from each participant before the study, and they were compensated upon completion. Data from four participants were excluded from analysis due to frequent movement (head or limbs) that affected data quality. Thus, data from a total of 24 participants (17 females; mean age: 22.23 ± 2.93 years) were included in subsequent analyses. A power analysis using G*Power 3.1 software indicated that a sample size of 24 was sufficient for a repeated measures *F*-test with a statistical power of 1–*β* = 0.80 and *α* = 0.05. Based on previous studies, the effect size for this study was expected to be medium, indicating good statistical power.

### 2.2 fNIRS data acquisition

**Figure 1:**
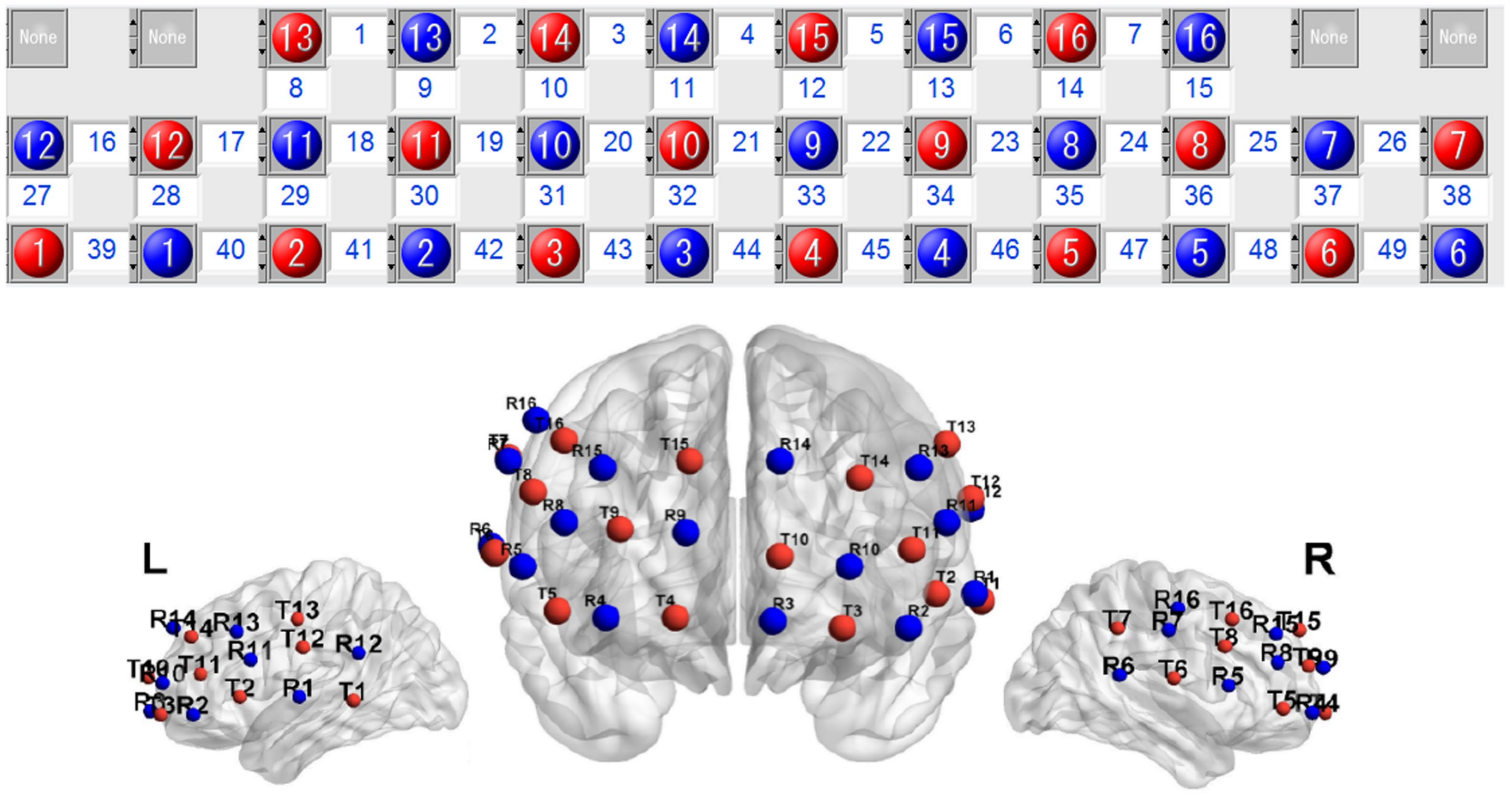
Schema for location of the optodes. Red circles indicate sources and blue circles indicate detectors.

The fNIRS system (OMM-2001, Shimadzu, Kyoto, Japan) with continuous wave laser diodes with wavelengths of 780, 805, and 830 nm was used to record cortical activities with a sampling rate of 19.61 Hz. A 49-channel system with 36 optodes, consisting of 16 light-source fibers and 16 detectors, was used and the interoptode distance was set at 3.0 cm. At the end of each experiment, a 3D locator (FASTRAK, Polhemus, Colchester, VT, USA) was used as an auxiliary tool to determine the Cz, Nz, AL, AR points, and probe positions. Probability registration was performed on the coordinates of each channel (MNI), and the corresponding Brodmann’s areas were identified using a brain template. The brain regions covered by each channel are shown in Figure 1.

### 2.3 Design and material

The experiment adopted a within-subject design. The independent variable was mismatch type with four levels: consistent, consonant mismatch, vowel mismatch, and tone mismatch conditions. The behavioral index was response time and accuracy, and the brain imaging index was oxygenated hemoglobin (HbO).

The experimental materials consisted of visual and auditory stimuli. The visual stimuli were 30 commonly used Chinese characters (with a frequency of more than 300 occurrences per million characters according to the modern Chinese word frequency list). Each visual Chinese character stimulus (e.g., “拔” pronounced ‘ba2’) was paired with four auditory stimuli, including one consistent (‘ba2’) with the character pronunciation and three mismatched in the consonant (‘ma2’), vowel (‘bo2’), and tone (‘ba4’), respectively. All auditory stimuli were correctly identified by 98.8% of participants not involved in the formal experiment. During the experiment, auditory stimuli were played through a speaker placed approximately 60 cm in front of the participants’ ears at a constant volume. All auditory stimuli were true syllables composed of a single consonant (b/p/m/f/d/t/n/l/g/k/h/j/q/x) and a single vowel (a/o/e/i/u). Consonants c/ch, s/sh, and r/y were not included to avoid confusion between flat and rolled tongue sounds. In the experiment, 30 items were balanced using a Latin square design to create three versions. In each version, each participant experienced only one experimental condition for each item. To ensure an equal number of consistent and mismatch trials, an additional 30 consistent stimuli were added, including 10 items under consistent conditions and 20 filler stimuli. Thus, each participant completed a total of 60 trials.

### 2.4 Experimental procedure

The fNIRS experiment uses a jitter design, which can not only prevent the participants from guessing the purpose of the experiment but also prevent the fatigue effect caused by the long experimental process (Schroeter et al., 2004). The instruction was presented to the participants at the beginning of the experiment, and 10 practice trials were performed after the participants understood the instruction to be familiarized with the procedure.

**Figure 2:**
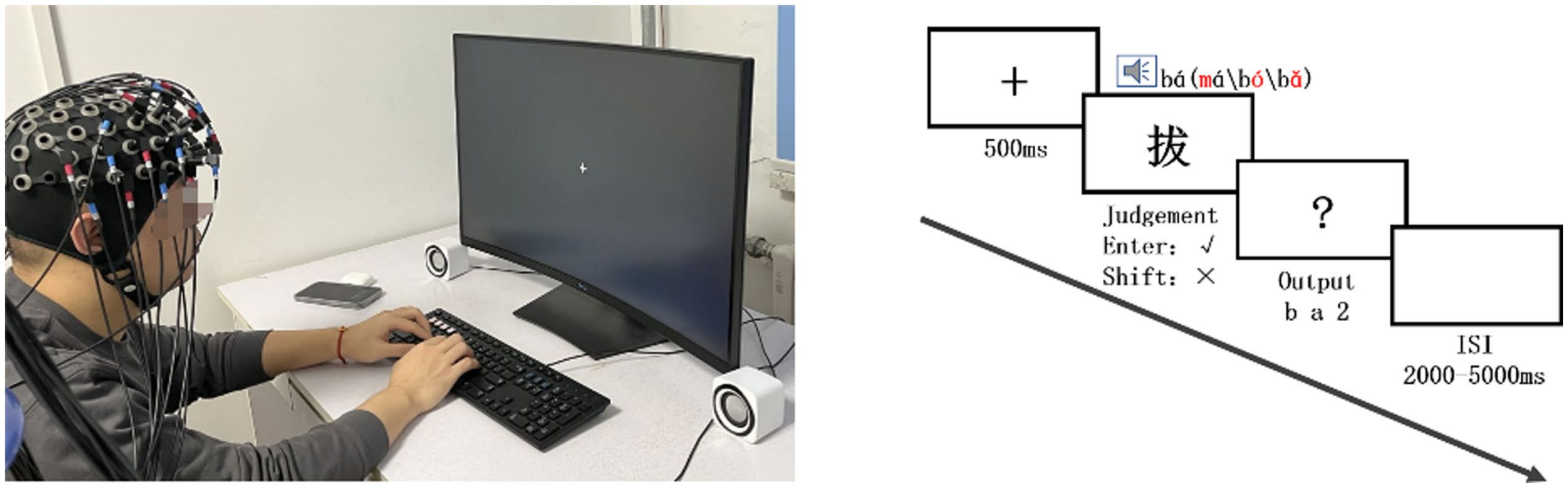
Scenario (left) and procedure (right).

The experimental scenario and procedure are shown in Figure 2. First, a fixation cross appeared for 500 ms in the center of the screen. After it disappeared, a white Chinese character (e.g., “拔” pronounced ‘ba2’, number 48 in bold) with a black background appeared in the center of the screen while an auditory stimulus (e.g., “ma2”) was presented through a speaker. Participants were asked to judge whether the pronunciation of the auditory and visual stimuli was consistent using the “Enter” and “Shift” keys on the right side of the keyboard (key balance across participants). Responses were made with the right hand to avoid differences in brain activation between the left and right hands. After the consistency judgment, a “?” appeared in the center of the screen, and participants were asked to name the Chinese character using a keyboard. The number keys 1–4 represented the four tones. All Chinese characters had only one consonant, one vowel, and no polyphonic characters, so the correct output was completed with three keystrokes. To avoid expectation effects, the 60 trials were presented in random order and separated by a jittered inter-stimulus interval (ISI) of 2000–5000 ms (Schroeter et al., 2004). The entire experiment (including wearing the optical cap and 3D positioning, etc.) lasted approximately 1 h.

### 2.5 Phonological awareness test

Before the formal experiment, all participants completed a phonological awareness test using a phoneme deletion task (Li and Shu, 2009; Cheng et al., 2015). In this task, participants were asked to report the pronunciation of characters with the consonant or vowel removed. Stimuli were true-character pinyin with 1–4 representing the four Mandarin tones. The test consisted of 40 items divided into three subtests: initial (consonant) deletion (e.g., kai1 as ai1), final (vowel) deletion (e.g., kai1 as k1), and middle (vowel) deletion (e.g., kuai1 as kai1).

During the test, participants were instructed to report the pronunciation content as quickly as possible while ensuring accuracy. The number of correct syllables and time taken were recorded. The number of correct syllables reported was divided by the time taken. This test is widely used in language research and has an internal consistency reliability ranging from 0.72 to 0.88 (Li and Shu, 2009; Cheng et al., 2015; Zhao et al., 2019).

### 2.6 Data analysis

In the output task, the subjects were asked to respond to each stimulus with three key presses. The three key presses represent the consonant, vowel, and tone of the visual word, respectively. According to a survey investigating subjects’ typing habits shortly after the experiment, subjects often have considered all the key-pressing steps before starting typing, rather than pressing one key and then thinking of the next step. Therefore, the time it took from the “?” appeared till the beginning of the first keypress was counted as the reaction time, which was in line with some current behavioral and EEG handwriting studies (He and Zhang, 2017; Wang and Zhang, 2021).

For behavioral data, SPSS (version 22.0) was used to analyze the accuracy and response time of the judgment task and the output task by one-way four levels repeated measures ANOVA. The four levels are consistent condition, consonant mismatch condition, vowel mismatch condition and tone mismatch condition, respectively. Post-hoc comparisons among the four conditions were conducted with Bonferroni correction.

fNIRS data were analyzed using the NIRS_SPM package in MATLAB (R2013b) (Jang et al., 2009), which modeled and analyzed blood sample data based on the general linear model (GLM). In this experiment, Δ [HbO] was used as the index for all data analyses due to its higher signal-to-noise ratio compared to Δ [HbR]. The original data were first analyzed using Principal Component Analysis (PCA) to remove global physiological noise (Cui et al., 2011). Hemodynamic Response Functions (HRF) were used to exclude noise caused by participants’ head movement, heartbeat, and breathing. Linear trends were then removed using high-pass (cutoff frequency: 0.01 Hz) filtering with the Wavelet-MDL method (Jang et al., 2009). Finally, HRF low-pass (cutoff frequency: 0.10 Hz) filtering was applied to remove physiological and machine noise.

GLM model fitting analysis was performed on the pre-processed HbO data. False response trials (2% in the judgment stage and 11% in the output stage) were first excluded. The beta for the four conditions was then imported into SPSS for a one-way within-subject repeated measures ANOVA with post-hoc test correction using the false discovery rate (FDR).

## 3 Results

### 3.1 Behavioral results

#### 3.1.1 Judgment process

**Figure 3:**
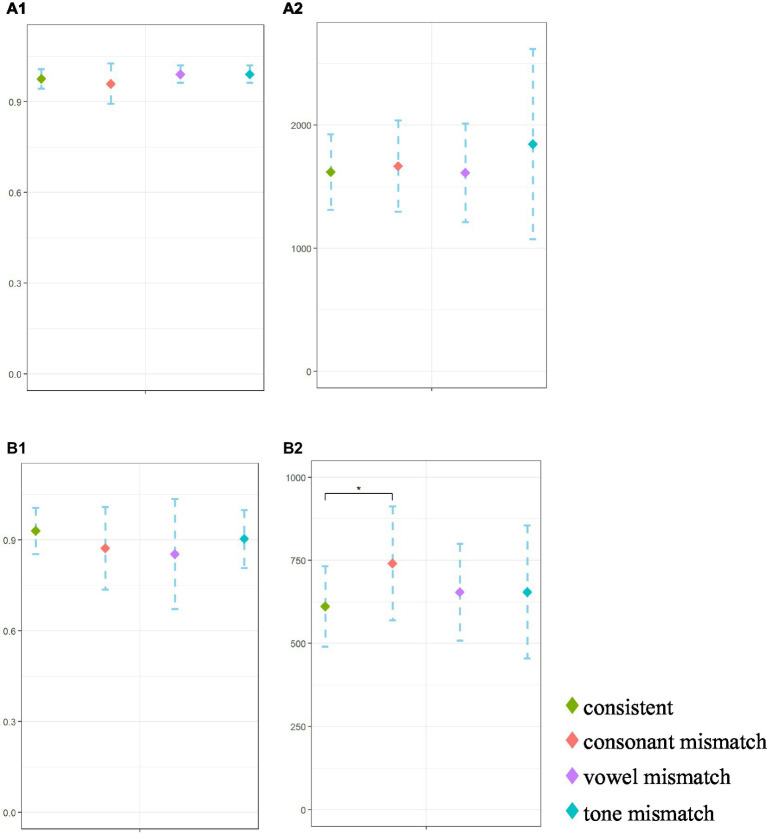
Behavioral results in judgment and output processes. **(A1)** Judgment ACC. **(A2)** Judgment RT. **(B1)** Output ACC. **(B2)** Output RT.

A one-way repeated measures ANOVA was used to analyze the accuracy ratio and reaction time in the judgment process. Results indicated a significant effect of mismatch type on the correct ratio, *F*(3,72) = 3.23, *p* = 0.046. However, post-hoc comparisons with Bonferroni correction showed no significant differences between any two of the four conditions (*ps* > 0.146). The main effect of mismatch type on reaction time was insignificant, *F*(3,72) = 2.33, *p* = 0.124. Results are shown in Table 1 and Figure 3.

**Table 1:** Behavioral results in judgment and output processes.

	Judgment				Output			
	C	CM	VM	TM	C	CM	VM	TM
ACC (*SD*)	0.98 (0.03)	0.96 (0.07)	0.99 (0.03)	0.99 (0.03)	0.93 (0.07)	0.87 (0.13)	0.85 (0.18)	0.90 (0.09)
RT (*SD*)	1619 (300)	1667 (363)	1613 (390)	1846 (756)	612 (118)	741 (168)	654 (142)	655 (194)

C, consistent condition; CM, consonant mismatch condition; VM, vowel mismatch condition; TM, tone mismatch condition.

#### 3.1.2 Output process

A repeated measures ANOVA showed no significant difference in the accuracy ratio among the four conditions, *F*(3,72) = 2.91, *p* = 0.068. However, when the reaction time was used as the indicator, the main effect of mismatch type was significant, *F*(3,72) = 5.73, *p* = 0.004, *η2 p* = 0.199. Post-hoc comparisons showed that reaction time in the consonant mismatch condition was significantly higher than in the consistent condition (*p* = 0.009), while there were no significant differences between the vowel mismatch and consistent conditions (*p* = 0.102) or between the tone mismatch and consistent conditions (*p* = 0.999). Results are shown in Table 1 and Figure 3.

### 3.2 fNIRS results

#### 3.2.1 Judgment process

A repeated measures ANOVA was performed on the beta of 49 channels in the judgment process. Results showed significant main effects of mismatch type in CH8, CH9, CH18, CH19, CH26, CH28, CH29, CH30, CH31, CH40, CH41, CH42, and CH43 (*Fs* > 3.10, *ps* < 0.05). Post-hoc comparisons using FDR correction (see Table 2) revealed that beta in Broca’s triangle of the left inferior frontal gyrus (CH29/CH41) were significantly lower in the consonant mismatch condition compared to the consistent condition. Additionally, beta in the left inferior frontal gyrus Broca’s triangle (CH29/CH30), parietal inferior frontal gyrus (CH31), superior temporal gyrus (CH40), and orbitofrontal cortex of the inferior frontal gyrus (CH42) were significantly lower in the consonant mismatch condition compared to the vowel mismatch condition.

**Table 2:** The fNIRS results of judgment process.

CH	MNI	Brodmann	*F*	*η^2^_p_*	*FDR* correction					
	(*x*,*y*,*z*)	area			C-CM	C-VM	C-TM	CM-VM	CM-TM	VM-TM
8	−56 5 42	BA6-L	3.57^*^	0.13	0.12	0.39	0.35	0.12	0.21	0.16
9	−47 34 36	BA45-L	3.99^*^	0.15	0.15	0.15	0.84	0.07	0.10	0.15
18	−53 29 28	BA45-L	3.10^*^	0.12	0.25	0.28	0.56	0.06	0.28	0.25
19	−41 54 22	BA46-L	5.67^**^	0.20	0.10	0.06	0.23	0.05	0.06	0.10
26	69–28 41	BA2-L	3.29^*^	0.13	0.16	0.51	0.18	0.17	0.17	0.51
28	−68 -13 25	BA43-L	3.84^*^	0.14	0.07	0.16	0.08	0.21	0.33	0.60
29	−60 17 19	BA44-L	4.41^**^	0.16	**0.04**	0.97	0.64	**0.04**	0.05	0.64
30	−51 44 10	BA45-L	4.62^**^	0.17	0.07	0.28	0.71	**0.03**	0.07	0.20
31	−35 64 6	BA10-L	5.49^**^	0.19	0.16	0.07	0.35	**0.03**	0.07	0.10
40	−64 1 8	BA48-L	3.89^*^	0.14	0.06	0.88	0.30	**0.03**	0.16	0.30
41	−56 32 1	BA45-L	3.73^*^	0.14	**0.03**	0.47	0.41	0.09	0.19	0.76
42	−44 56–5	BA11-L	4.68^*^	0.17	0.12	0.15	0.81	**0.04**	0.12	0.12
43	−24 69–4	BA11-L	2.97^*^	0.11	0.65	0.12	0.12	0.12	0.19	0.51

C, consistent condition; CM, consonant mismatch condition; VM, vowel mismatch condition; TM, tone mismatch condition.

**p < 0.01, *p < 0.05 (The *p*-values are FDR corrected). Bold: *p*-value < 0.05.

#### 3.2.2 Output process

Repeated measures ANOVA showed significant main effects of mismatch type in CH18, CH19, CH28, CH29, CH30, CH31, CH40, and CH41 (*Fs* > 3.31, *ps* < 0.05). Post-hoc comparisons using FDR correction (see Table 3) revealed that compared to the consistent condition, the beta was lower in the left inferior frontal gyrus Broca’s triangle (CH29/CH41) and left superior temporal gyrus (CH40) in the consonant mismatch condition. In contrast, the beta was higher in the left middle frontal gyrus (CH19) and left inferior frontal gyrus (CH31) in the vowel and tone mismatch conditions.

**Table 3:** The fNIRS results of output process.

CH	MNI			Brodmann	*F*	*η^2^_p_*	FDR correction					
				area			C-CM	C-VM	C-TM	CM-VM	CM-TM	VM-TM
	*x*	*y*	*z*									
18	−53	29	28	BA45-L	3.31^*^	0.13	0.21	0.21	0.21	0.06	0.40	0.21
19	−41	54	22	BA46-L	3.69^*^	0.14	0.44	**0.03**	**0.04**	0.10	0.14	0.30
28	−68	−13	25	BA43-L	3.51^*^	0.13	0.08	0.08	0.12	0.43	0.43	0.98
29	−60	17	19	BA44-L	5.48^**^	0.19	**0.02**	0.63	0.12	**0.02**	**0.04**	0.38
30	−51	44	10	BA45-L	3.94^*^	0.15	0.10	0.36	0.72	0.07	0.13	0.33
31	−35	64	6	BA10-L	3.59^*^	0.13	0.87	**0.04**	**0.04**	**0.04**	0.37	0.28
40	−64	1	8	BA48-L	4.63^*^	017	**0.05**	0.88	0.08	**0.05**	0.10	0.19
41	−56	32	1	BA45-L	4.21^*^	0.14	**0.04**	0.48	0.10	0.10	0.10	0.48

C, consistent condition; CM, consonant mismatch condition; VM, vowel mismatch condition; TM, tone mismatch condition.

**p < 0.01, *p < 0.05 (The *p*-values are FDR corrected). Bold: *p*-value < 0.05.

**Figure 4:**
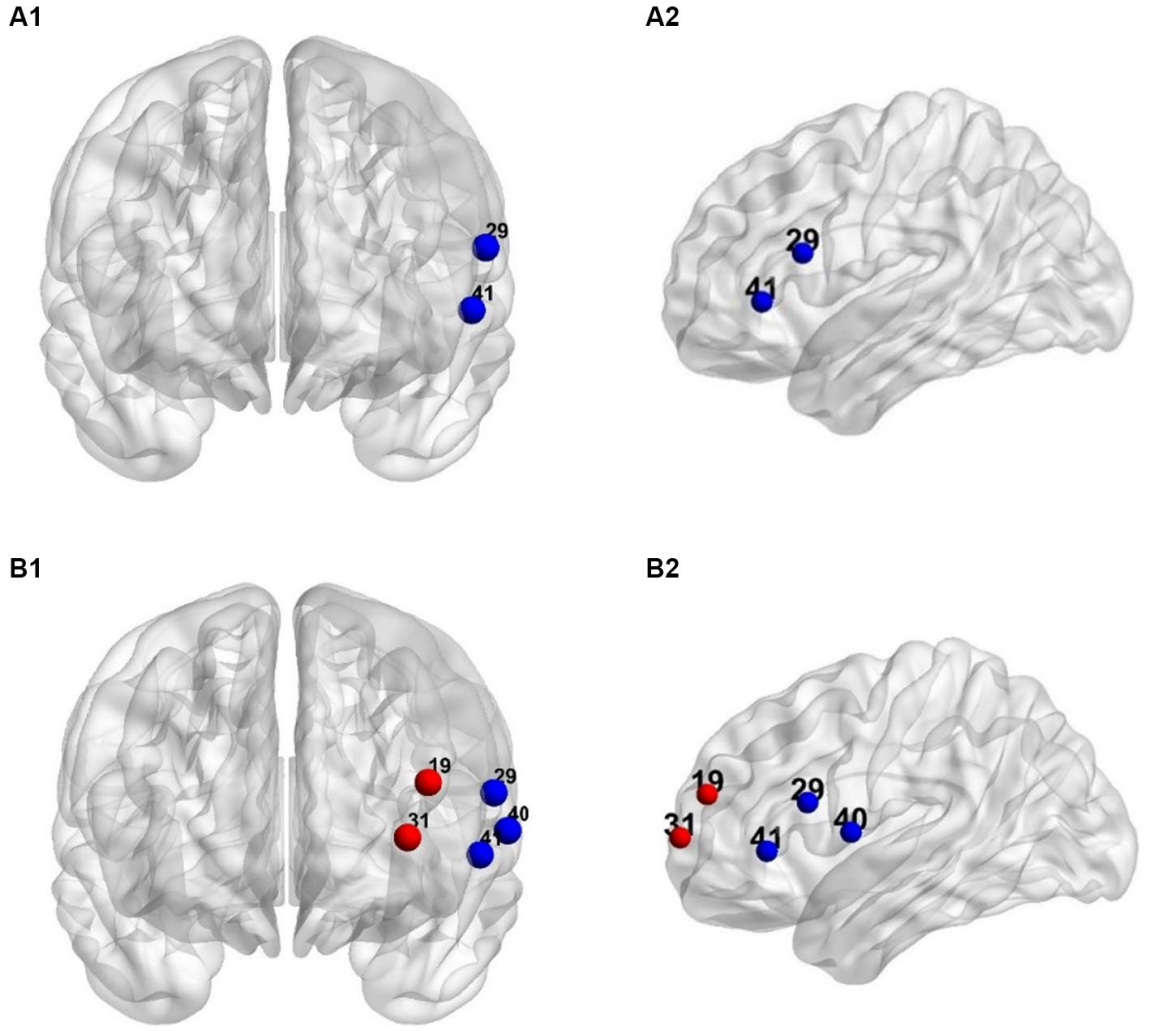
The schematic of brain activation for the judgment and output processes. The dots in the figure indicate channels that significant differences between mismatch condition and consistent condition after *post hoc* comparisons (*p*s < 0.05). Blue dots: The beta of consonant mismatch condition is significantly lower than that of consistent condition. Red dots: The beta of vowel and tone mismatch condition is significantly higher than that of consistent condition. **(A1)** Front view of judgment. **(A2)** Left view of judgment. **(B1)** front view of output. **(B2)** Left view of output.

In addition, the beta in the inferior frontal gyrus Broca’s triangle (CH29), parietal inferior frontal gyrus (CH31), and superior temporal gyrus (CH40) were significantly lower in the consonant mismatch condition compared to the vowel mismatch condition. The beta for consonant mismatch was also significantly lower than for tone mismatch in Broca’s triangle of the inferior frontal gyrus (CH29). However, no significant difference was found between the vowel and tone mismatch conditions. A schematic of brain activation for the judgment and output processes is shown in Figure 4.

#### 3.2.3 Whole process

Analysis of fNIRS data during the entire experiment (judgment process + output process) showed a significant main effect of mismatch type in the top of the left inferior frontal gyrus (CH31), *F*(3,72) = 3.22, *p* = 0.04. However, post-hoc tests using FDR correction did not find significant differences between any two conditions (*ps* > 0.05).

### 3.3 Correlation analysis

**Figure 5:**
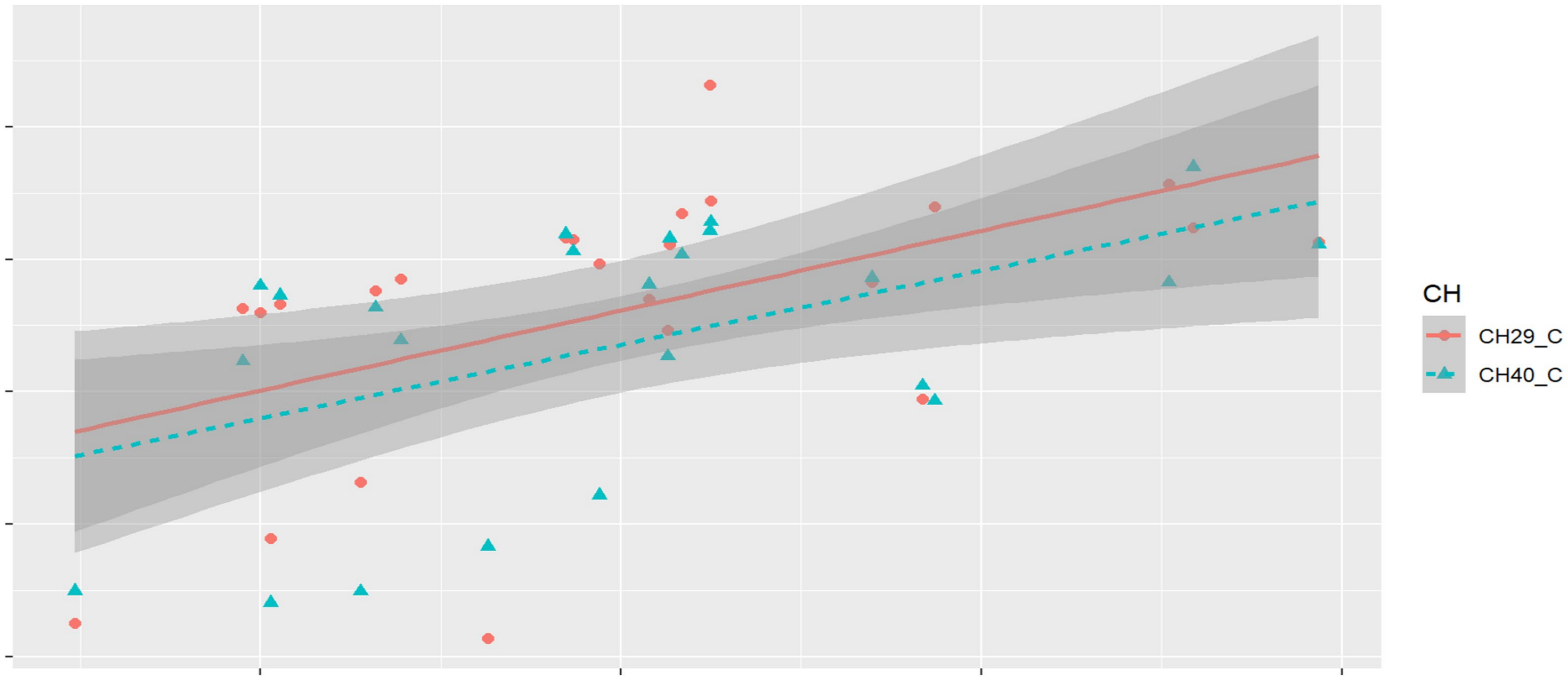
Schematic diagram of correlating results of phonological awareness score rate and HbO activation beta.

The time taken for the phonological awareness test varied greatly (*M* = 122.96 s, *SD* = 31.13). Therefore, score rate (correct quantity/time) was used as a measure of phonological awareness achievement. Channels with significant differences were identified as channels of interest (CH19, CH29, CH31, and CH40). The correlation between the phonological awareness score rate and the beta for conditions with significant differences in channels of interest was then calculated. Results using FDR correction showed significant positive correlations between the phonological awareness score rate and the beta values for the consonant condition in CH29 (*r* = 0.53, *p* = 0.03) and CH40 (*r* = 0.51, *p* = 0.02), as shown in Figure 5.

## 4 Discussion

This study investigated the role and neural mechanisms of consonant, vowel, and tone information in Chinese production using fNIRS technique. We adopted a word-word interference paradigm with four conditions: consistent, consonant mismatch vowel mismatch, and tone mismatch conditions. Results showed that consonant mismatch condition activated Broca’s triangle in the left inferior frontal gyrus (CH29/CH41) and the left superior temporal gyrus, while both vowel and tone mismatch condition activated the left middle frontal gyrus (CH19) and parietal inferior frontal gyrus (CH31).

Our findings regarding the brain regions involved in consonant, vowel, and tone processing during Chinese word production consistent with some previous studies. For example, an fMRI study by Liu et al. (2006) showed that changes in vowels and tones induced activation in the bilateral inferior frontal gyrus, anterior central gyrus, and insula. Siok et al. (2003) found that the left inferior frontal gyrus was involved in consonant processing. Gandour et al. (2003) found that vowels evoked greater activation than consonants and tones in the middle frontal gyrus. These results are in line with our findings regarding the neural mechanisms underlying vowel and tone processing during Chinese speech processing, indicating that the brain regions identified in our study are reliable. Our findings suggested that the left inferior frontal gyrus, left middle frontal gyrus, and left superior temporal gyrus are involved in Chinese production.

Although the role and neural mechanisms of segmental and tonal information in Chinese processing have been extensively studied, most research has focused on speech perception or recognition (Ye and Connine, 1999; Gandour et al., 2003; Tong et al., 2008; Li et al., 2010, 2013, 2014; Hu et al., 2012; Zhao et al., 2016; Choi et al., 2017; Zou et al., 2020; Zhang et al., 2021; Chang and Hsieh, 2022), while few has examined the role of segmental and tonal information in speech production (Liu et al., 2006; Wong and Chen, 2008, 2009, 2015; Zhang and Damian, 2009; Wang et al., 2015; Wiener and Turnbull, 2016). To the best of our knowledge, only one study has systematically investigated the processing of consonant, vowel, and tone during Cantonese spoken word production in a PWI paradigm (Wong and Chen, 2008). The results of Wong and Chen (2008) showed that when the auditory distractors shared the same vowels as the visual targets, the response time for a naming task was significantly faster than for irrelevant stimuli. However, the same effect was not observed for consonants or tones. Therefore, it was concluded that vowels play a more crucial role in speech production. Wong and Chen (2008) further found that the combination of “consonant + vowel” had a greater facilitation effect than “vowel + tone,” leading to the conclusion that consonants have a greater effect than tones. However, our behavioral results showed that only the reaction time in the consonant mismatch condition was significantly higher than in the consistent condition, suggesting a stronger role of consonant than vowel and tone in Mandarin word production. This difference between this finding and Wong and Chen’s study may be related to the output mode. Specifically, in our study, pinyin typing was used as the output mode, which may emphasize the role of the first output phoneme (consonant) in Chinese pinyin. Thus, our behavioral results only showed the role of the onset (consonant). Moreover, the inconsistent results may also be related to different indices, as the fNIRS results of the present study showed that consonant, vowel, and tone all play a role in Chinese language output.

The present study furthered our understanding of the role of segmental and suprasegmental information during Chinese word processing. As segments and tones are characterized by different acoustic properties, comparison between the relative roles and the processing of tone and segment has long been a focus of psycholinguistics. Specifically, listeners distinguish among consonants through rapidly changing bursts and formant transitions (Tong et al., 2008), while vowel is distinguished by steady-state formant frequencies (Tong et al., 2008). Tone is distinguished by pitch contour and pitch level (Tong et al., 2008; Tsang et al., 2011; Wang et al., 2013; Liang and Du, 2018). Moreover, in pinyin, there is a clear separation between consonant and vowel (Liu et al., 2006), with consonant always preceding vowel (Gandour et al., 2003). Tone is typically carried by the vowel during spoken words production (Ye and Connine, 1999), so the two often appear simultaneously. Given the unique acoustic cues for tone perception and the temporal relation between tone and segments in speech production, uncovering how tone is activated and its roles relative to segments during Chinese word processing will ultimately lead to more complete word processing models in tonal languages.

However, few studies have systematically manipulated consonant, vowel, and tone in their experimental designs. Some studies either never distinguish between consonant and vowel (Brown-Schmidt and Canseco-Gonzalez, 2004; Schirmer et al., 2005; Zhang and Damian, 2009) or compared only one of them with tone (Liu et al., 2006; Luo et al., 2006; Hu and Xu, 2011; Hu et al., 2012; Li et al., 2014; Choi et al., 2017; Zou et al., 2020; Zhang et al., 2021). In contrast, the present study further separated consonant and vowel as segmental information to provide a more complete picture of the comparisons among consonant, vowel, and tone processing during Chinese word production.

Although some previous studies often studied consonant and vowel as a whole (Brown-Schmidt and Canseco-Gonzalez, 2004; Schirmer et al., 2005; Zhang and Damian, 2009; Li et al., 2013; Sereno and Lee, 2015), our results showed differences in neural activation between these two types of phonemes during Chinese typing. Consonant-sensitive brain regions (CH29/41) were located in the left inferior frontal gyrus near the temporal lobe and the left superior temporal gyrus. Vowel-sensitive regions (CH31/19) were found in the superior part of the frontal gyrus and the left middle frontal gyrus in the left hemisphere. These findings suggest a spatial dissociation in the neural basis underlying consonant and vowel production. Differences in processing consonants and vowels were also observed in activation patterns: Compared to the consistent condition, consonant mismatch induced lower HbO activation while vowel mismatch induced higher HbO activation. Further examination of the differences between consonant and vowel mismatch conditions revealed that in the left inferior frontal gyrus Broca’s triangle (CH29), parietal inferior frontal gyrus (CH31), and superior temporal gyrus (CH40), HbO activation in the consonant mismatch condition was significantly lower than in the vowel mismatch condition. An interesting finding of our study is that the tone mismatch condition also induced higher HbO activation than the consistent condition and significantly higher HbO activation than the consonant mismatch condition in Broca’s triangle of the left inferior frontal gyrus (CH29). In summary, the neural correlates of consonant processing differed from vowel and tone, which were similar to each other.

The findings of the present study were in line with some previous studies focusing on the effects of consonant and vowel on language output (Gandour et al., 2003; Tong et al., 2008; Wong and Chen, 2008; Zhang et al., 2011; Wiener and Turnbull, 2016; Zhao et al., 2016). For example, in a study by Wiener et al. (2016), participants were asked to reconstruct a pseudoword into a real word by changing the consonant, vowel, tone, or making a free choice. Results showed that compared to the consonant change condition, accuracy was significantly lower and reaction time significantly higher in the vowel change condition. This suggests that vowel plays a more important role than consonant in constraining word identity. Similar results were observed in Tong et al. (2008) using a speeded classification paradigm, which showed a significantly higher interference effect for vowel compared to consonant. Based on those previous researches, our study further highlights a dissociation in neural basis underlying consonant and vowel processing for language output.

However, compared with consonant processing, tone processing is more similar to vowel both in activated brain regions and mode of activation. This indicates that similar neural mechanisms were involved in tone and vowel processing during Chinese word production. Previous studies in Chinese speech processing showed that vowel and tone were processed as an integrated unit at the early stage of spoken word perception (Choi et al., 2017) and they evoked comparable neural responses during spoken sentence comprehension (Schirmer et al., 2005). The present study found additional evidence of integrated processing of vowel and tone in typing. Although the tone information is the fundamental frequency (F0), the tone in Chinese language production still induces the activation of the left hemisphere, which is associated with language processing. This is in contrast to non-tonal language, in which tonal processing is more likely to activate brain regions on the right hemisphere associated with music processing (Blumstein and Cooper, 1974; Mazzucchi et al., 1981). The involvement of left hemisphere in processing tonal information found in this study might be attributed to the tonal-language nature of Chinese that tone affects semantic processing and plays a critical role in language production.

It is undeniable that language production, regardless of picture naming or text naming, inevitably accompanies comprehension. Specifically, language production starts from concept activation, and only when individuals understand the concepts of the stimuli they receive can they generate language or text. Therefore, whether there are differences in the brain networks related to language comprehension and production has always attracted considerable attention of researchers. Previous studies on spoken language production have found that despite some overlap in the neural networks of language comprehension and production, there are differences in their associated brain areas (Haller et al., 2005; Golestani et al., 2006; den Ouden et al., 2008; Grande et al., 2012; Humphreys and Gennari, 2014; Pylkkanen et al., 2014; Matchin and Hickok, 2016; Takashima et al., 2020). However, no consensus has yet been reached on the specific brain areas that reflect the differences between language comprehension and production. A meta-analysis study (Walenski et al., 2019) found that the left middle frontal gyrus, left posterior Middle Temporal Gyrus, and lateral occipital cortex were activated in language production tasks but not left inferior frontal gyrus (LIFG), which is an area essential for language comprehension (Snijders et al., 2009; Fedorenko et al., 2012; Matchin et al., 2017; Zaccarella et al., 2017). Nevertheless, another meta-analysis study found that compared to language comprehension, LIFG has stronger activation in language production (Indefrey, 2018). Together, those findings led to the belief that there are certain differences in the brain networks involved in language production and understanding (Giglio et al., 2022). Although this study focuses on the neural mechanisms of consonants, vowels, and tones in language production, it also examines the subjects’ comprehension process through judgment tasks. The results showed that some brain areas (LIFG Broca’s triangular area: CH29/41) are involved in both language comprehension and typing, indicating that language production is accompanied by language comprehension. Moreover, we also found unique brain areas (left middle frontal gyrus: CH19, top of left inferior frontal gyrus: CH31, and left superior temporal gyrus: CH40) associated with Chinese typing, indicating certain differences between the neural mechanisms underlying language understanding and production.

It is worth noting that although interference paradigms have been widely used in speech production studies, the exact cognitive processes involved in this paradigm might be more complicated than auditory word perception and target word production. For instance, perceiving incongruent auditory words might also evoke error detection, response inhibition, decision-making, etc. However, those processes might only play a minor role in the target word typing in the current study for the following reasons. First, some existing studies showed that the brain regions involved in executive processes are mostly concentrated in the hippocampus and cingulate gyrus. For example, error detection was found to occur in the cingulate gyrus (Ullsperger and von Cramon, 2001, 2004; Garavan, 2002; Fassbender et al., 2004; Ridderinkhof et al., 2004; Chevrier and Schachar, 2010). Response inhibition was observed to occur in the cingulate gyrus (Botvinick et al., 2004; Fan et al., 2005; Aron, 2016; Hung et al., 2018), and brain regions related to decision making are in the dorsal anterior cingulate cortex and right superior temporal sulcus (Román et al., 2019). However, the results of this study show that the brain regions associated with the consonant mismatch condition are in the left inferior frontal gyrus, Broca’s triangular area, and the left superior temporal gyrus. The brain regions associated with the vowel and tone mismatch conditions are in the left middle frontal gyrus and the top of the left inferior frontal gyrus, which are different from the brain regions associated with executive processes. Therefore, the results of this study did not provide strong evidence of the involvement of executive processes during Chinese word typing under the interference paradigm. Secondly, a large number of existing studies using interference paradigms (whether picture or word interference) have shown that the results of consonant, vowel, and tone mismatch stimulus conditions essentially reflect the phonological facilitation effect on phonological encoding rather than the inhibitory effect by the incongruent stimuli as in Stroop task (Wong and Chen, 2008, 2009, 2015; Wang et al., 2018; Wong et al., 2018; Zhang et al., 2022). In summary, the auditory spoken words in the current study should mainly evoke phonological facilitation effects instead of conflict resolution during target word production.

In addition, individual differences are ubiquitous in language processing but are often treated as errors in most theories and experimental methods (Kidd et al., 2018). However, psycholinguistic theories need to account for individual differences or at least be applicable to different individuals (Kidd et al., 2018). Therefore, our study explored individual differences in phoneme processing during language output. We measured each participant’s phonological awareness and found a significant positive correlation between phonological awareness and beta for the consonant mismatch condition. This suggests that individuals with strong phonological awareness also have better processing ability for segmental information. Thus, phonological awareness can predict an individual’s ability to process segmental information in language output. Many previous studies have also suggested that segmental information plays a greater role in language production than tone (Zhang, 2008; Zhang and Damian, 2009; Wang et al., 2015). As such, phonological awareness is more likely to manifest in segmental information processing, which has much higher information value than tonal information in spoken word processing. Based on this, we hypothesized that an individual’s phonological awareness can predict their language output to some extent, with this predictive ability being more evident in segmental than tonal information processing. Yet, this conclusion requires future research to validate.

To summarize, the present study focused on Chinese typing, which is increasingly common in today’s society, and explored the role of tonal and segmental information in typing Chinese words. This enriches existing research that has mostly focused on spoken and handwritten language production. Like handwriting, typing is learned and developed and represents another form of human language output, similar to sign language for hearing-impaired individuals. With advances in technology, typing and handwriting have become equally important in school education (Mangen and Balsvik, 2016; Amez and Baert, 2020; Verhoeven et al., 2020). Previous research has found that handwriting enhances orthographic awareness while typing enhances phonological awareness and phonetic-semantic mapping in Chinese learning (Chen et al., 2016a,b; Guan and Wang, 2017). In addition, a review of case studies on handwriting and typing impairment found that both can serve as important indicators of stroke (Sharma et al., 2019). Therefore, it is necessary to study the neural mechanisms of typing as an increasingly important mode of language output. Based on previous research, our study investigated the role and neural mechanisms of segmental and suprasegmental information in the typing process. Results showed that consonant processing has a different neural basis from vowel and tone processing, which involve the same brain regions. Based on previous studies in speech output (Liu et al., 2006; Zhang and Damian, 2009), our results further highlighted the important role of tone during Chinese typing.

However, it should be noted that our study required participants to name Chinese characters, which may have introduced interference from the font shape of the characters. It is also unclear whether participants actively extracted semantic information from the characters. Future research could consider using picture naming to investigate the role of consonant, vowel, and tone in more active semantic extraction. Additionally, our study only recorded the time and brain signal for the first key press during participants’ typing. Although most current handwriting studies also use the first stroke as an indicator (He and Zhang, 2017; Wang and Zhang, 2021), this may have emphasized the role of consonants. Future research could record the entire language output process to more objectively investigate the processing mechanisms of consonant, vowel, and tone. Furthermore, most of the existing phonemic awareness tests are conducted offline. To get a complete picture of individual differences in language production, future studies could design a more suitable online method for phonemic awareness testing and collect neural data during the phonemic awareness testing process for further analysis. It is worth mentioning that as the same visual stimuli were paired with different auditory syllables under different conditions, it is possible that the processing of different auditory stimuli across conditions might confound the results. This issue should be addressed in future research. Finally, although typing plays an increasingly important role in daily life, little is known about the neural mechanisms underlying typing. It is crucial for future researchers to conduct a systematic comparison among typing and speech production, handwriting, and even the use of sign language to reveal the similarities and differences among those language production processes.

## 5 Conclusion

Our study investigated the role and neural basis of consonant, vowel, and tone during Chinese typing using fNIRS techniques. Results showed that the consonant, vowel and tone all play a role in language production. Further examination revealed spatial separation and differences in HbO activation patterns between consonants and vowels. In contrast, brain regions activated by tone and vowel overlapped spatially and had similar activation patterns.

## Data availability statement

The raw data supporting the conclusions of this article will be made available by the authors, without undue reservation.

## Ethics statement

The studies involving humans were approved by Northeast Normal University, School of Psychology. The studies were conducted in accordance with the local legislation and institutional requirements. The participants provided their written informed consent to participate in this study. Written informed consent was obtained from the individual(s) for the publication of any potentially identifiable images or data included in this article.

## Author contributions

JY: Data curation, Software, Visualization, Writing – original draft, Writing – review & editing. YZ: Writing – review & editing. YW: Funding acquisition, Methodology, Resources, Supervision, Writing – review & editing.
